# Tibetan Plateau increases the snowfall in southern China

**DOI:** 10.1038/s41598-023-39990-x

**Published:** 2023-08-07

**Authors:** Liping Wang, Haijun Yang

**Affiliations:** 1 Department of Atmospheric and Oceanic Sciences, School of Physics, Peking University, Beijing, 100871 China; 2 Department of Atmospheric and Oceanic Sciences and CMA-FDU Joint Laboratory of Marine Meteorology, Fudan University, 2005 Songhu Road, Shanghai, 200438 China; 3 Shanghai Scientific Frontier Base for Ocean-Atmosphere Interaction Studies, Fudan University, Shanghai, 200438 China

**Keywords:** Climate sciences, Climate and Earth system modelling

## Abstract

The role of the Tibetan Plateau (TP) in Asian hydrological climate is crucial, yet there is a lack of quantitative estimates regarding its impact on snowfall in China. Some opinions suggest that the TP functions as a large barrier that obstructs cold outbreaks, protecting southern China from severe snowstorms. Through topography experiments with and without the TP, our study suggests that the TP's presence results in a 60% decrease in snowfall in northern China by significantly reducing moisture. In contrast, it promotes a 1500% increase in snowfall in southern China, particularly from November to March, by drawing cold air from the north and moisture from the south to southern China. The presence of TP significantly enhances winter relative humidity in southern China, causing discomfort for humans. This research refutes some trending views and improves our understanding of the TP's role in China's winter climate.

## Introduction

As the world’s highest and largest plateau, the Tibetan Plateau (TP) plays a vital role in Asian hydrological climate, including arid central Asian and moist South and East Asia^1–5^. Paleoclimatic evidence shows that China experienced a drastic climate transformation during the Cenozoic (the past 66 million years ago, Ma), from an extensive zonal arid belt to monsoon-dominant pattern^6,7^ (about 22 Ma); the timing of such change agrees well with the significant TP uplift (about 25–20 Ma)^8,9^.

Previous studies investigated comprehensively the impact of the TP on Asian hydrological climate. The TP contributes to the dry climate in northwest China by blocking the moisture from the south and to the wet climate in southern China by strengthening the Asian monsoon^10–12^. In summer, the TP increases monsoon precipitation in southern China^11,13–15^; in winter, it has a dominant effect on lee cyclogenesis and associated northerly cold surges^16^.

There are still some issues unsettled. First, the controversy on TP’s role in winter climate in China remains. Some studies claimed that the TP’s uplift, especially the northern part, blocks the northerly winter monsoon to southern China, increasing precipitation over southern China^15,17,18^. Others reported that the TP’s presence intensifies the winter monsoon over southern China, but showing different winter precipitation in the region: increase^19^, decrease^20^, or trivial change^13^. Second, most studies explored TP’s role in China’s climate qualitatively from a monsoon perspective. There is a lack of quantitative estimate on TP’s influence on wintertime snowfall in China. All these lead to some trending views mainly among weather forecasters and self-media: the TP can protect southern China from severe cold events and snowstorm because the TP is an obstacle for cold air from the north. In other words, if the TP were not present in Asian continent, the cold air would move southward easily to reach southern China, leading to more snowfall there. This point of view sounds intuitive at first look, but actually has serious scientific flaws: if the TP were not there, the planetary circulation in the mid-latitudes would be more zonal, which would prevent the cold air from invading southern China.

In this paper, we quantify TP’s role in China’s snowfall and cold surges, aiming to clarify some related issues. Idealized orography experiments with and without the TP are carried out to investigate the impact of TP’s presence on snowfall in China, especially in southern China. This paper is organized as follows: In section "Model and simulations", the model and experiments are described. In section "Results", snowfall response to TP perturbation is demonstrated, and its mechanisms are revealed. Summary and discussion are provided in section “Summary and discussion”.

## Model and simulations

The experiments performed in this study use the National Center of Atmospheric Research’s Community Earth System Model version 1.0 (CESM1.0). This is a widely used fully-coupled global climate model, consisting of an atmosphere model (Community Atmosphere Model: CAM4)^21^, land surface model (Community Land Model: CLM4)^22^, sea ice model (Community Ice Code: CICE4)^23^, ocean model (Parallel Ocean Program: POP2)^24^, and a coupler (CPL7). We employ the low-resolution configuration (T31_gx3v7) in CAM4 with an atmospheric grid of roughly 3.75°$\times$3.75° and 26 vertical levels. The CLM4 has the same horizontal resolution as the CAM4. The POP2 has 60 levels in the vertical, a uniform 3.6° spacing in the zonal direction and a non-uniform spacing in the meridional direction; specifically, it has 0.6° resolution near the equator, gradually increasing to the maximum 3.4° at 35°N/S, and then decreasing poleward. The CICE4 shares the same horizontal grid as the POP2. No flux adjustments are used in CESM1.0.

Two groups of experiments with different orography were conducted (Fig. S1). The first group includes a 2400-yr control run (named “Real”) and a 400-yr sensitivity run without the TP orography (named “NoTibet”). Real retains all the world’s present-day topography^25^ (Fig. S1a). NoTibet starts from year 2001 of Real, with the orography around the TP flattened to 50 m above mean sea level (Fig. S1b), and is then integrated for 400 years to reach the equilibrium state. The second group includes a 1200-yr control run with a global flat topography set to 50 m (named “Flat”) (Fig. S1c) and a 400-yr sensitivity run with only the TP set to its realistic topography and the other continental regions remained at 50 m (named “OnlyTibet”) (Fig. S1d). OnlyTibet starts from year 801 of Flat and is integrated for 400 years. Except for topography, all other boundary conditions, including vegetation, soils and other surface characteristics remain the same as those in CTRL. We apply a preindustrial CO_2_ level of 285 ppm. These experiments are single orography sensitivity tests rather than paleoclimate simulation experiments in which one needs to prescribe several geologic boundary conditions simultaneously. The climate responses with and without the TP are obtained by subtracting the results of Flat (NoTibet) from those of OnlyTibet (Real). We focus on quasi-equilibrium responses, which are averaged over the last 100 years of model integrations. We use the monthly model outputs including temperature, wind, sea level pressure, relative humidity, geopotential height and vertical velocity. Figure S2 shows the winter precipitation, surface winds and surface air temperature from our control simulation and observations (GPCP data and ERA5 reanalysis). It is clearly shown that the control run can well simulate the mean climate in China.

In this paper, we focus on winter (December-February, DJF) climate responses. The snowfall data comes from model outputs: the sum of convective snow water equivalent and large-scale snow water equivalent, depending on precipitation and temperature. Precipitation that occurs below freezing temperature (0℃) is considered snow.

Moisture content is the total column water vapor, representing the precipitable water. It can be written as:

1 $$Q=\frac{1}{g}\underset{1000\,hPa}{\overset{300\,hPa}{\int }}q dp$$

where q is the specific humidity and g is the acceleration of gravity. It is vertically integrated from 1000 hPa to 300 hPa, as the water vapor above 300 hPa is negligible.

Moisture transport is calculated as follows^26^:

2 $$MT=\frac{1}{g}\underset{1000\,hPa}{\overset{300\,hPa}{\int }}\left(\overrightarrow{V}q\right) dp$$

where $\overrightarrow{V}$= (*u, v*) is the horizontal wind. The divergence of moisture transport can be divided into two terms^27^:

3 $$\nabla \cdot MT=\frac{1}{g}\underset{1000\,hPa}{\overset{300\,hPa}{\int }}\nabla \cdot \left(\overrightarrow{V}q\right) dp= \frac{1}{g}\underset{1000\,hPa}{\overset{300\,hPa}{\int }}\overrightarrow{V}\cdot \nabla q dp+ \frac{1}{g}\underset{1000\,hPa}{\overset{300\,hPa}{\int }}q\left(\nabla \cdot \overrightarrow{V}\right) dp$$

The first term on the right is the moisture advection and the second term on the right represents the contribution of wind divergence. The divergence (convergence) in the wind field, like an anomaly anticyclonic (cyclonic) circulation, corresponds to the divergence (convergence) in the moisture transport field, which contributes to less (more) moisture^28^.

To have a more comprehensive understanding of the TP’s contribution on the winter climate in different regions of China, we divide China into five regions: northeast China (110°–132°E, 40°–53°N), north China (110°–120°E, 34°–40°N), northwest China (73°–110°E, 34°–45°N), southeast China (110°–120°E, 21°–34°N), and southwest China (97°–110°E, 21°–34°N). We perform student’s *t*-test to examine statistical significance of all analyses. Most changes over China exceed the 95% significance level, which is expected because altering the TP topography induces strong mechanical forcing, and should cause strong responses around the globe. For visual clarity, we do not show significance test in most figures.

## Results

### Snowfall response and its mechanism

**Figure 1:**
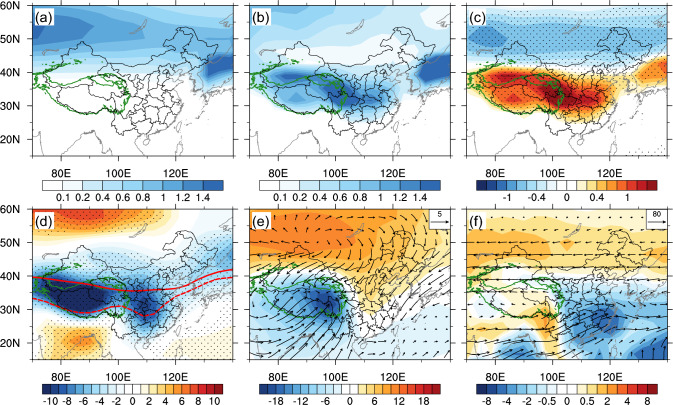
Snowfall (units: cm/year) in (**a**) Flat, (**b**) OnlyTibet and (**c**) its change in OnlyTibet, with respect to Flat. Changes in (**d**) surface temperature (shading; units: °C), (**e**) surface wind (vector; units: m/s) and sea-level pressure (shading; units: hPa) in OnlyTibet, with respect to Flat. Solid and dashed red curves in (**d**) represent the temperature zero-line in Flat and OnlyTibet, respectively. (**f**) shows changes in vertically integrated moisture transport (vector; units: $\mathrm{kg }{\mathrm{m}}^{-1} {\mathrm{s}}^{-1}$) and its divergence (shading; units: ${10}^{-5}\mathrm{ kg }{\mathrm{m}}^{-2} {\mathrm{s}}^{-1}$) in OnlyTibet, with respect to Flat. All values are averaged over the boreal winter (DJF). The TP region is marked by enclosed green contour. Stippling in (**c**,**d**) represents the variables changes are significant at the 95% confidence level based on the student *t-*test. All maps were generated using open source software NCL (NCAR Command Language) Version 6.6.2 (https://www.ncl.ucar.edu/Download/).

The presence of the TP leads to snowfall increase in southern China by ~1500% during the boreal winter (DJF) (Fig. 1c). In Flat, snowfall distributes mainly north of 40°N, and is negligible in southern China (Fig. 1a). After adding the TP, the center of maximum snowfall shifts southward remarkably (Fig. 1b). By contrast, snowfall in Xinjiang, Inner Mongolia and northeast China is reduced by ~60%. Therefore, the presence of the TP promotes snowfall in southern China. Note that the results from the two groups of experiments are alike (Figs. 1c vs S3), suggesting trivial effects from the other topographic features. For the sake of succinct discussion, we only use Flat and OnlyTibet experiments in the following sections.

To understand the snowfall response to the TP perturbation, we begin by figuring out the condition for snowfall formation, which is mainly controlled by temperature condition (below 0°C) in wintertime^29–31^. Moisture supply is a necessary condition. It does not depend much on vertical movement, as water vapor can be condensed without lifting under low-temperature conditions.

First, the presence of the TP leads to significant cooling over China in winter, similar to the finding in previous studies^19,32,33^. The temperature zero-line (red curves in Fig. 1d) shifts southward by 10°–15° latitude-wise, suggesting the TP can significantly draw cold air from the north to southern China, and thus promoting snowfall there. Change in surface air temperature (SAT) is mainly caused by anomalous northerly wind (Fig. 1e). The TP blocks the westerlies in the lower level and generates an “asymmetric dipole” pattern, with a large anticyclonic circulation to the north, enhancing the Siberian high^34^. The anomalous northerly wind in the east of the anticyclonic circulation is favourable for the cold air transport to southern China. In addition, a strong cooling appears over the TP region, resulting mainly from the lapse rate effect in the troposphere in response to the TP uplift. Most changes in snowfall and SAT are significant at 95% confidence level, suggesting the robust impact of the TP on China’s wintertime climate. For visual clarity, we do not show significance test in other figures in this work.

**Figure 2:**
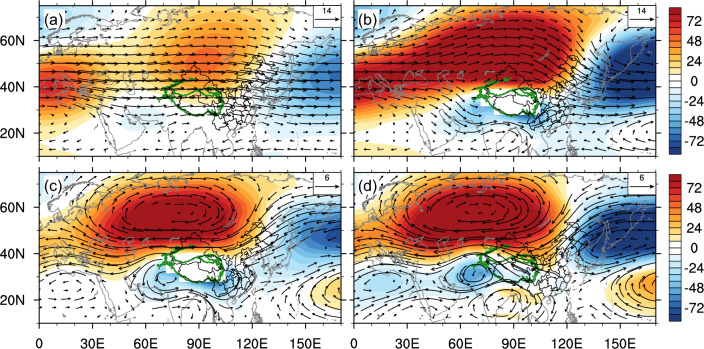
Wind (vector; m/s) and geopotential height (shading; m) at 700 hPa averaged over boreal winter in (**a**) Flat, (**b**) OnlyTibet and (**c**) their changes in OnlyTibet, with respect to Flat. (**d**) is the same as (**c**), but at 500 hPa. The TP region is marked by enclosed green contour. The geopotential height is obtained by subtracting its zonal-mean value.

Second, the presence of the TP increases moisture content over southern China. In Flat, moisture is transported by the zonal westerlies from the Atlantic to China, diverging (converging) over southern (northern) China (Fig. S4a). Adding the TP leads to more moisture transported from the Atlantic to Indian Ocean, converging over southern China (Fig. S4b), and thus promoting snowfall formation there. Changes in atmospheric circulation in the lower level are responsible for moisture transport and convergence. Adding the TP generates an anomalous cyclonic circulation to the south of the TP, which brings moisture from the tropics northward to southern China, converging over southern China and leading to snowfall increase (Fig. 2c). This meridional moisture transport disappears in Flat due to the more zonal westerlies (Fig. 2a). Meanwhile, reduced westerlies due to the TP presence leads to less moisture transport toward northern China. There is an anomalous anticyclonic circulation causing moisture divergence, reducing water-vapor content over northern China (Fig. 1f). This dry condition is not conducive for snowfall formation even though the winter temperature in northern China is very low in winter.

Third, the presence of the TP provides favourable large-scale circulation condition for snowfall over southern China. The TP promotes winter cold surges in China, since it facilitates the establishment of the Siberian high, blocking high and ridge, which are necessary for cold air outbreaks reaching the south. Cold advection in front of the Siberian high is conducive to triggering cold surges^35–38^. Blocking high can prevent the exchange of cold air from high latitudes and warm air from low latitudes, and promote the accumulation of strong cold air^39–42^. Ridge is the key for southward transport of cold air^43,44^. After adding the TP, the meridional motion increases due to the deflection of the westerlies, triggering the establishment of a trough near 90°E and a ridge near 130°E (Fig. 2b). The Siberian high also increases due to a stronger sea-land thermal contrast. Moreover, prominent enhancement of the blocking high related to the TP appears (Fig. 2d). Therefore, the TP plays an important role for cold air outbreaks and snowfall in China. Without the TP, the planetary-scale winds would be roughly zonal; and there would be no trough and ridge for southward transport of cold air (Fig. 2a). In addition, the TP contributes to the establishment of the southern trough (Fig. 2c). Warm and moist air drawn by the southern trough meets cold and dry air drawn by the ridge over southern China, thereby greatly promoting snowfall in southern China^45–47^.

### Regional snowfall response

**Figure 3:**
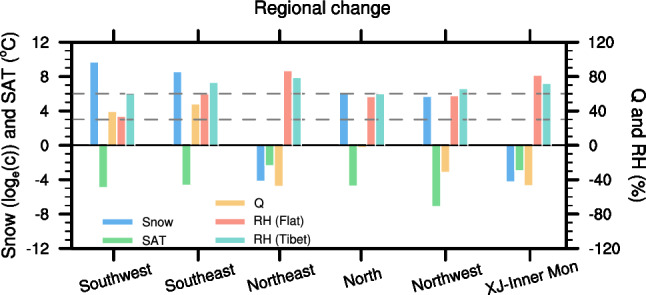
Bar chart for regional snowfall change (units: ${\mathrm{log}}_{e}(c)$), moisture content change (Q; units: %), surface air temperature (SAT) change (units: ℃), and relative humidity (RH; units: %) in Flat and OnlyTibet experiments over southwest China, southeast China, northeast China, north China, northwest China, and Xinjiang-Inner Mongolia. Left ordinate is for snowfall and SAT and right ordinate is for Q and RH. For snowfall, we use the natural log ordinate ${\mathrm{log}}_{e}(c)$, *c* is the relative snowfall change (units: %) in OnlyTibet, with respect to Flat. For example, if ${\mathrm{log}}_{e}(c)=10$, $c={e}^{10}\%\approx 22000\%$, which means the snowfall is increased by 220 times; if ${\mathrm{log}}_{e}(c)=-4$, $c={-e}^{4}\%\approx -55\%$, which means the snowfall is decreased by 55%. The grey dashed lines represent comfortable RH levels of 30% and 60%, respectively.

We quantify TP’s role in snowfall, cold air and moisture conditions in different regions of China. Figure 3 shows the changes of snowfall, temperature and moisture in each region. The TP significantly promotes snowfall in southeast China and southwest China, since it contributes greatly to moisture increase and temperature decrease in these two regions. Although adding the TP reduces water-vapor content over north China and northwest China, the remarkably lowered temperature still facilitates atmospheric water-vapor condensation and increases snowfall in these regions. Only Xinjiang and Inner Mongolia (73°–110°E, 40°–47°N) experience snowfall decrease thanks to sharply declined moisture content. Similarly, northeast China has less snowfall due to dramatic reduction of moisture content.

**Figure 4:**
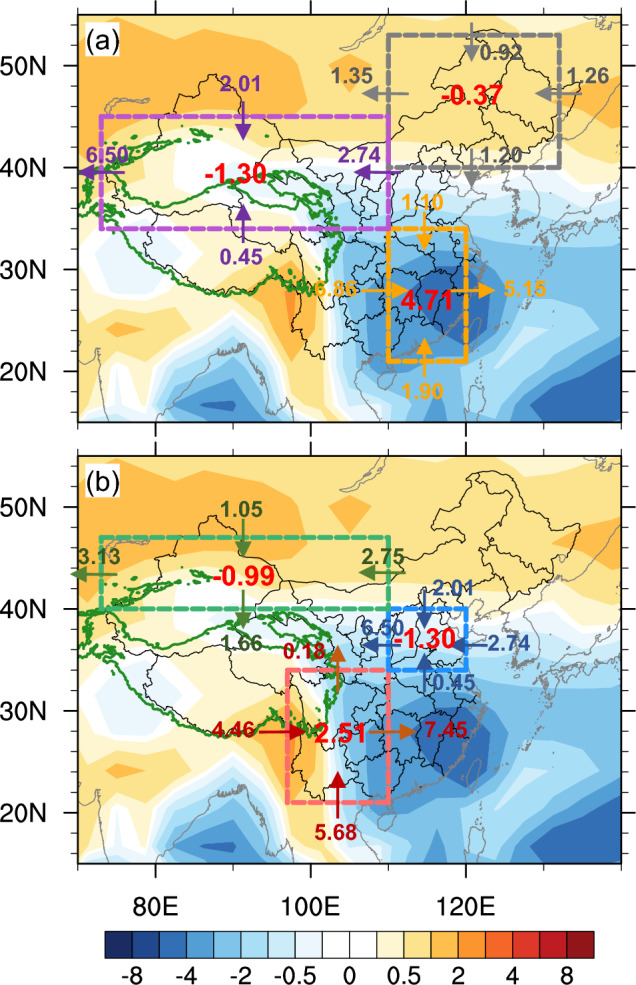
Changes in regional moisture transport (units: ${10}^{7}\mathrm{ kg }{\mathrm{s}}^{-1})$ and its budget (numbers). Shading represents vertically integrated moisture divergence. Positive (negative) value in red denotes eastward and northward (westward and southward) transport. Numbers at boundaries of rectangles denote transports across the boundaries. (**a**) Boxes in purple, grey and yellow represent northwest China (73°–110°E, 34°–45°N), northeast China (110°–132°E, 40°–53°N) and southeast China (110°–120°E, 21°–34°N), respectively. (**b**) Boxes in green, blue and red represent Xinjiang and Inner Mongolia (73°–110°E, 40°–47°N), north China (110°–120°E, 34°–40°N) and southwest China (97°–110°E, 21°–34°N), respectively.

To better recognize TP’s contribution to regional moisture budget, we calculate the moisture flux across each rectangle boundary of each region (Fig. 4). The TP increases the net moisture flux by about $4.71\times {10}^{7} \mathrm{kg}/\mathrm{s}$ (78.37%, compared with Flat) over southeast China (Fig. 4a, yellow box), by drawing moisture mainly from the Indian Ocean and Pacific Ocean (western pathway of $6.86\times {10}^{7} \mathrm{kg}/\mathrm{s}$ (322.07%) and southern pathway of $1.90\times {10}^{7} \mathrm{kg}/\mathrm{s}$ (27.38%)). The enhanced meridional circulation leads to more meridional moisture convergence over southeast China. The net moisture flux over southwest China is $2.51\times {10}^{7} \mathrm{kg}/\mathrm{s}$ (72.75%) (Fig. 4b, red box), which mainly comes from the west and south, that is, from the Indian Ocean. The anomalous northeasterly wind (Fig. 2c) transports moisture from the Pacific to north China and northeast China via eastern and northern pathways, but more moisture exports from western and southern boundaries, eventually leading to a net moisture loss of about $-1.30\times {10}^{7} \mathrm{kg}/\mathrm{s}$ (−213.11%) (Fig. 4b, blue box) and $-0.37\times {10}^{7} \mathrm{kg}/\mathrm{s}$ (−32.17%) (Fig. 4a, grey box), respectively. In northwest China (Fig. 4a, purple box), expect for the moisture from the Pacific, about $0.45\times {10}^{7} \mathrm{kg}/\mathrm{s}$ (30.41%) moisture flux comes in from the southern trough, indicating that the TP drives moisture to the south of northwest China. By contrast, the TP blocks the moisture transport from low-latitude oceans to Xinjiang and Inner Mongolia, as there is about $1.66\times {10}^{7}\mathrm{ kg}/\mathrm{s}$ (−80.58%) moisture flux exporting to the south (Fig. 4b, green box), leading to a drier climate and less snowfall.

Relative humidity (RH) is a key factor for human comfort; and quantifying TP’s contribution to regional RH has practical significance. A comfortable RH level in winter is about 30–60%, which is recommended by Heating, ventilation and air conditioning (HVAC) design standards^48–50^. In Flat, RH is lower in the mid-latitudes due to suppressed deep convection by downward motion in the subtropical high-pressure belt. It increases from mid to high latitudes due to lowered temperature and to moisture advection from the subtropics^51^ (Fig. S5a). Without the TP, the RH over most regions of China would be at a comfort level except for northeast China, Xinjiang and Inner Mongolia (Fig. 3). Adding the TP leads to a dramatic increase in RH over southern China, especially southwest China (Fig. 3), suggesting that the presence of the TP would not help provide human comfort.

### Monthly snowfall response

**Figure 5:**
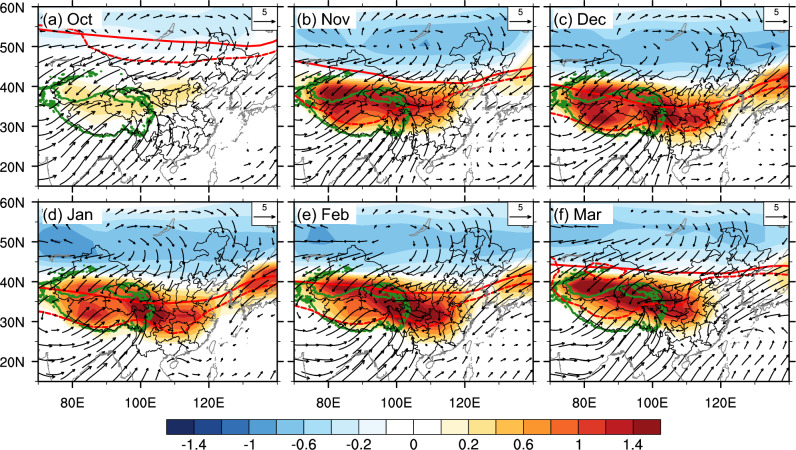
Monthly changes in snowfall (shading; units: cm/year) and surface wind (vector; units: m/s) in OnlyTibet, with respect to Flat. Solid and dashed red lines represent temperature zero-line in Flat and OnlyTibet, respectively. The TP region is marked by enclosed green contour. Vectors less than 0.1 m/s in magnitude are omitted for clarity.

Figure 5 displays monthly snowfall responses to TP presence. Adding the TP leads to temperature zero-line shifting southward significantly to southern China during December-February; combined with the enhanced moisture from the Indian Ocean (Fig. S6), this promotes snowfall in southern China. In March and November, temperature zero-line moves southward to northern China, enhancing the snowfall there. The snowfall response is mainly attributed to monthly temperature change (Fig. 5, red curve), as the snowfall change is consistent with the southward shift of temperature zero-line.

The monthly location of temperature zero-line after adding the TP depends on two factors. One is the seasonal variation of background temperature zero-line (Fig. 5, solid red curve), which moves southward in winter and retreats northward in summer. The other is the seasonal change in surface circulation after the TP is added. The TP is located in the strong westerly belt during December-January; and the deflection of the westerlies generates strong anomalous high to the north of the TP, indicating the establishment of blocking high and ridge, which transports cold air southward to southern China. Additionally, the anomalous low to the south of the TP draws moisture northward to southern China, enhancing the snowfall there. In March and November, the anomalous high is weakened, so cold air transports can only reach northern China, promoting snowfall in the north.

## Summary and discussion

In this study, we quantify TP’s role in winter snowfall in China by conducting experiments with and without the TP. We conclude that the presence of the TP promotes the snowfall in southern China and reduces snowfall in northern China. Snowfall change is mainly controlled by low-temperature condition (below 0℃). Adding the TP intensifies the winter northerly monsoon, drawing cold air southward to southern China. The presences of blocking high, Siberian high and ridge are conducive for cold air outbreaks. Additionally, the anomalous southerlies bring moisture northward to southern China, helping promote snowfall there. The TP facilitates snowfall in southern China mainly during November-March, as the temperature zero-line moves southward to southern China. In northern China, the anomalous anticyclonic circulation facilitates moisture divergence, causing less snowfall.

This study corrects trending views from some self-media platforms and weather forecasters, where people think that the TP acts as a giant wall to block cold air from the north, thus keeping southern China from cold surges and snowfall. The westerly trough, representing cold air, is weakened when it moves southward to the TP, so the cold air cannot blow southward directly but alters the path to northeast China; that is, if the TP were not there, the cold air would move southward to southern China. Our experiments show an opposite result. Without the TP, the planetary-scale air flow over China is more zonal, so there would be no ridge and trough transporting cold air and moisture to southern China.

The findings in this study can help us correctly estimate TP’s contribution to China’s winter climate. Previous studies paid more attention to TP’s impact on summer precipitation in China from the perspective of summer monsoon, rather than on winter snowfall. Although these sensitivity experiments are highly idealized, they provide quantitative estimates of TP’s role in China’s winter climate.

The results obtained from our simulations are consistent with previous research findings in some aspects. For example, the enhanced Siberian high, ridge and downstream trough responses to TP’s presence show resemblance to previous results^13,20,52^. Previous studies revealed that the uplift of Tibetan Plateau results in a reduced temperature in East Asia^16,53^, which is similar to our studies. Furthermore, some studies using atmosphere model demonstrated that the existence of the TP enhances the winter cold air outbreak to East Asia, as well as the southerly winds towards southern China, which facilitates the transport of moisture, forming persistent rainfall in late winter and early spring over southern China. These findings align with our conclusions^33,54,55^. Kutzbach et al. showed an increase wintertime snow cover throughout much of Eurasian, including southern China in a world with the TP uplift using atmospheric circulation model. However, we show snowfall decreased over the area north of 40°N. This is possibly because we use coupled models with full ocean dynamics, which can better study the role of TP on Asian wintertime climate. We understand that the conclusions drawn in this study may be model-dependent. For example, the resolutions for atmosphere and ocean model components are rather coarse, which cannot well capture the realistic topography and precipitation and may cause biases in snow water equivalent. With such limitations in mind, different models with high resolutions and better designed experiments are needed in the future to truly understand the role of the TP in China’s winter climate.

### Supplementary Information

Supplementary Information.

## Data Availability

Datasets from this research can be obtained at https://doi.org/10.5281/zenodo.7463796.
