# Supplementary material for: Tibetan Plateau increases the snowfall in southern China
Source: Sci Rep. 2023 Aug 7;13:12796. doi: 10.1038/s41598-023-39990-x (PMC10406920; doi:10.1038/s41598-023-39990-x)
Supplement: Supplementary file 1 — Supplementary Information. [file 41598_2023_39990_MOESM1_ESM.docx]

***Scientific Reports***

Supporting Information for

**Tibetan Plateau increases the snowfall in southern China**

Liping Wang^1^ and Haijun Yang^2,3*^

*^1^Department of Atmospheric and Oceanic Sciences, School of Physics, Peking University, Beijing, 100871, China.*

*^2^Department of Atmospheric and Oceanic Sciences and CMA-FDU Joint Laboratory of Marine Meteorology, Fudan University, Shanghai, 200438, China.*

*^3^Shanghai Scientific Frontier Base for Ocean-Atmosphere Interaction Studies, Fudan University, Shanghai 200438, China.*

*Corresponding author:*

Haijun Yang ([yanghj@fudan.edu.cn)](mailto:yanghj@fudan.edu.cn))

**Contents of this file**

Figures S1 to S6


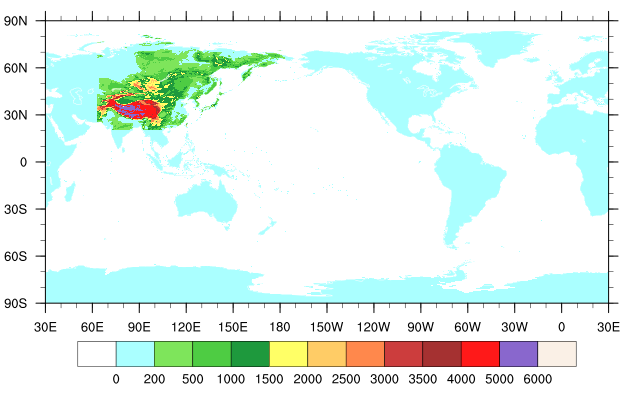

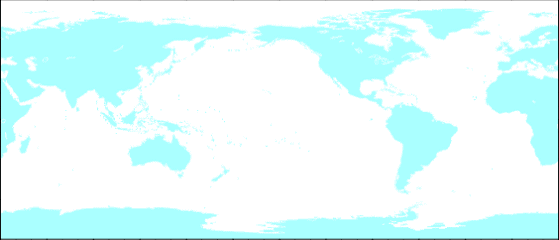

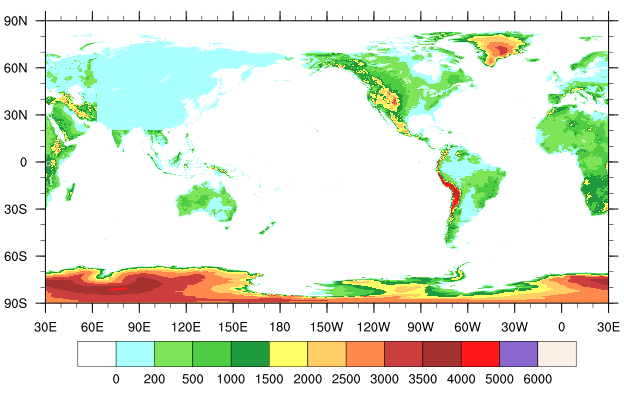

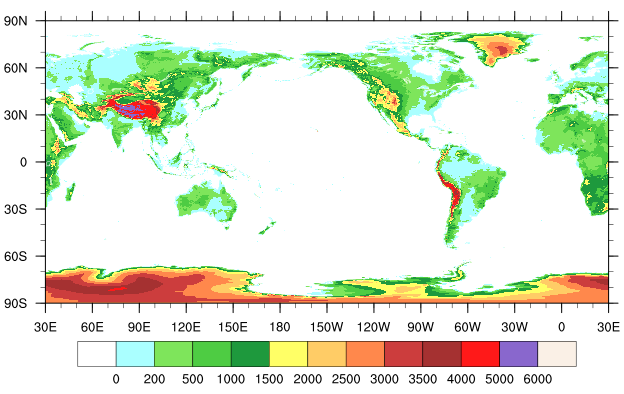


(a) Real

(b) NoTibet

(c) Flat

(d) OnlyTibet


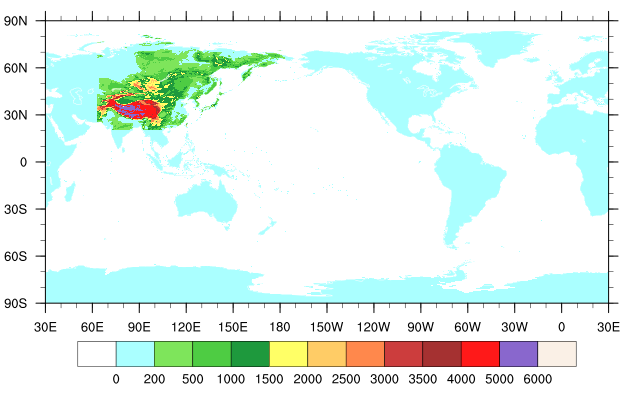

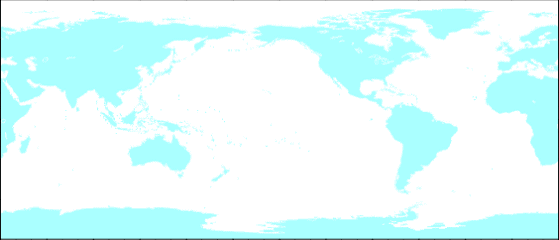

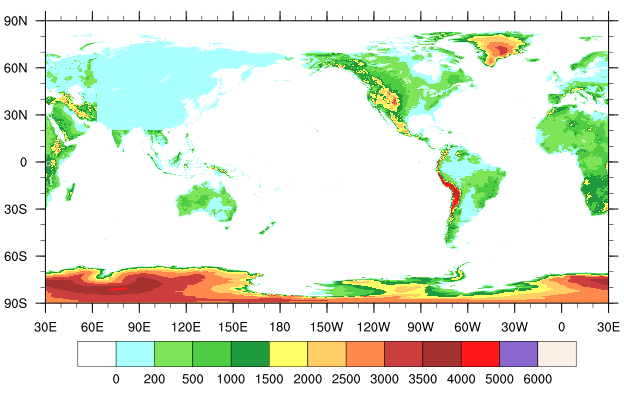

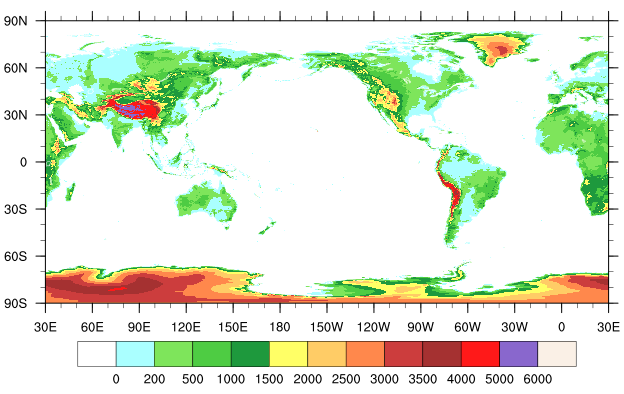


(a) Real

(b) NoTibet

(c) Flat

(d) OnlyTibet

Figure S1. Topography configuration in coupled model experiments. (a) Realistic topography used in Real (the control run), (b) modified topography without the Tibetan Plateau (TP) used in NoTibet, (c) modified topography with flat global topography used in Flat, and (d) modified topography with only TP topography used in OnlyTibet. Maps were generated using open source software NCL (NCAR Command Language) Version 6.6.2 (<https://www.ncl.ucar.edu/Download/>).

(a)

(b)

(c)

(d)

Figure S2. Precipitation (units: cm/year) from (a) GPCP data and (b) CESM control run. Surface wind (vectors; units: m/s) and surface air temperature (shading; units: °C) from (c) ERA5 reanalysis and (d) CESM control run. All variables are averaged over boreal winter (December-January-February). Observations are averaged between 1979 and 2020. CESM control run is averaged over the last 100 model year. Maps were generated using open source software NCL (NCAR Command Language) Version 6.6.2 (<https://www.ncl.ucar.edu/Download/>).

(a)

(b)

(c)

Figure S3. Snowfall (units: cm/year) averaged over the boreal winter (DJF) in (a) NoTibet, (b) Real and (c) its changes in Real, with respect to NoTibet. Maps were generated using open source software NCL (NCAR Command Language) Version 6.6.2 (<https://www.ncl.ucar.edu/Download/>).

(a)

(b)

Figure S4. Vertically integrated moisture transport (vector; units: $\mathbf{kg}\mathbf{m}^{\boldsymbol{-}\mathbf{1}}\mathbf{s}^{\boldsymbol{-}\mathbf{1}}$) and its divergence (shading; units: $\mathbf{10}^{\boldsymbol{-5}}\mathbf{kg}\mathbf{m}^{\boldsymbol{-2}}\mathbf{s}^{\boldsymbol{-}\mathbf{1}}$) averaged over the boreal winter (DJF) in (a) Flat and (b) OnlyTibet, respectively. Maps were generated using open source software NCL (NCAR Command Language) Version 6.6.2 (<https://www.ncl.ucar.edu/Download/>).

(a)

(b)

(c)

Figure S5. Relative humidity (units: %) averaged over the boreal winter (DJF) in (a) Flat, (b) OnlyTibet and (c) its changes in OnlyTibet, with respect to Flat. Maps were generated using open source software NCL (NCAR Command Language) Version 6.6.2 (<https://www.ncl.ucar.edu/Download/>).

(a) Oct

(b) Nov

(c) Dec

(d) Jan

(e) Feb

(f) Mar

Figure S6. Monthly changes in vertically integrated moisture transport (vector; units: $\mathbf{kg}\mathbf{m}^{\boldsymbol{-}\mathbf{1}}\mathbf{s}^{\boldsymbol{-}\mathbf{1}}$) and its divergence (shading; units: $\mathbf{10}^{\boldsymbol{-5}}\mathbf{kg}\mathbf{m}^{\boldsymbol{-2}}\mathbf{s}^{\boldsymbol{-}\mathbf{1}}$) in OnlyTibet, with respect to Flat. Shown are October to March maps. Maps were generated using open source software NCL (NCAR Command Language) Version 6.6.2 (<https://www.ncl.ucar.edu/Download/>).
